# Ultra-wide bandgap semiconductor Ga_2_O_3_ power diodes

**DOI:** 10.1038/s41467-022-31664-y

**Published:** 2022-07-06

**Authors:** Jincheng Zhang, Pengfei Dong, Kui Dang, Yanni Zhang, Qinglong Yan, Hu Xiang, Jie Su, Zhihong Liu, Mengwei Si, Jiacheng Gao, Moufu Kong, Hong Zhou, Yue Hao

**Affiliations:** 1 State Key Discipline Laboratory of Wide Bandgap Semiconductor Technology, School of Microelectronics, Xidian University, Xi’an, 710071 China; 2 Department of Electronic Engineering, Shanghai Jiao Tong University, Shanghai, 200240 China; 3 State Key Laboratory of Electronic Thin Films and Integrated Devices of China, University of Electronic Science and Technology of China, Chengdu, 61005 China

**Keywords:** Electrical and electronic engineering, Electronic devices

## Abstract

Ultra-wide bandgap semiconductor Ga_2_O_3_ based electronic devices are expected to perform beyond wide bandgap counterparts GaN and SiC. However, the reported power figure-of-merit hardly can exceed, which is far below the projected Ga_2_O_3_ material limit. Major obstacles are high breakdown voltage requires low doping material and PN junction termination, contradicting with low specific on-resistance and simultaneous achieving of n- and p-type doping, respectively. In this work, we demonstrate that Ga_2_O_3_ heterojunction PN diodes can overcome above challenges. By implementing the holes injection in the Ga_2_O_3_, bipolar transport can induce conductivity modulation and low resistance in a low doping Ga_2_O_3_ material. Therefore, breakdown voltage of 8.32 kV, specific on-resistance of 5.24 mΩ⋅cm^2^, power figure-of-merit of 13.2 GW/cm^2^, and turn-on voltage of 1.8 V are achieved. The power figure-of-merit value surpasses the 1-D unipolar limit of GaN and SiC. Those Ga_2_O_3_ power diodes demonstrate their great potential for next-generation power electronics applications.

## Abstract (web summary)

The simultaneous achievement of high breakdown voltage and low resistance is a contradictory point because it would require high and low doping simultaneously. Here, Zhou et al. achieve a power figure-of-merit of 13.2 GW/cm2 through hole injection and conductivity modulation effect.

## Introduction

Advanced semiconductor material holds great promise of providing higher conversion efficiency as well as maintaining higher voltage for modern industrial- and consumer-scale power electronics. Ultra-wide bandgap (UWB) semiconductor with general bandgap (*E*_g_) greater than 4 eV can sustain a higher critical field (*E*_c_) and hence a higher blocking voltage is achievable at a smaller resistance and power electronic component dimension, which turns out to be more efficient than its narrow bandgap material Si and wide bandgap material GaN and SiC counterparts, as summarized in Table 1 of Supplementary Information. The general concept lies behind is that the high electric-field (*E*) and high temperature driven of the electron excitation from valance band to conduction band is inherently suppressed by the UWB. Therefore, power electronics based on UWB materials are spontaneously endowed with high breakdown voltage (BV) at a lower material thickness and resistance. Combined with good mobility (*μ*), a crucial power device parameter Baliga’s figure-of-merit (B-FOM~*μ* × *E*_c_^3^) of UWB semiconductors could be several or tens of folds of those wide bandgap materials GaN and SiC as well as more than thousands of times of narrow bandgap material Si^1,2^. However, it should be noted that the major tyranny of the UWB forbids achieving effective both n- and p-type doping simultaneously. Among those intriguing UWB semiconductor materials, the emerging Ga_2_O_3_ is now regarded as one of the most promising materials for next-generation high-power and high-efficiency electronics, due to its cost-effective melt-grown large-scale and low defect density substrate as well as the controllable n-type doping^3^.

Ga_2_O_3_ with *E*_g_ = 4.6–4.8 eV, high *E*_C_ = 8 MV/cm and decent intrinsic *μ* = 250 cm^2^/Vs has yielded a B-FOM to be around 3000, which is four times GaN and ten times SiC. Being the mainstream of the UWB semiconductor, Ga_2_O_3_-based power electronics are expected to bring higher blocking voltage at a lower specific on-resistance (*R*_on,sp_) for power switching applications. Tremendous efforts have been dedicated to explore the material property and push the device limit, and hence significant progresses are acquired during the past 5 years. Despite those intriguing achievements, it should be noted that those performances especially the representative device parameter power figure-of-merit (P-FOM = BV^2^/*R*_on,sp_) are much inferior to the projected material limit, or even cannot be comparable with the 1-D unipolar limit of the GaN and SiC^4,5^. Like other UWB semiconductors with the difficulties of achieving both highly conductive p- and n-type materials at the meantime, one of the major obstacles is the lack of p-type Ga_2_O_3_ which can be utilized as the PN homo-junction termination for the BV improvement. It was calculated that shallow acceptor does not exist and it was also predicted that the holes are self-trapped inherently^6^. As a result, unipolar Ga_2_O_3_ power electronics dominate most of the research and few reports are available about the bipolar transport study. Due to the challenge of realizing p-type Ga_2_O_3_ on lightly-doped n-type Ga_2_O_3_ layer, the BV of the vertical Ga_2_O_3_ power diodes was limited, although various types of edge termination (ET) methods were employed^7^. On the other hand, some wide-bandgap p-type materials like NiO_*x*_ with *E*_g_ of 3.8–4 eV and Cu_2_O with *E*_g_ ~ 3 eV, controllable doping and decent hole mobility of 0.5–5 cm^2^/V s turn out to be a good counterpart of p-type Ga_2_O_3_ to boost the diodes performance^8,9^. The combination of p-NiO_x_ and n-Ga_2_O_3_ is a feasible route for the Ga_2_O_3_ development, and the recent progress of the Ga_2_O_3_ PN hetero-junction (HJ) diodes shows a P-FOM of 1.37 GW/cm^2^, which is comparable to the P-FOM value of state-of-the-art Schottky barrier diodes (SBDs)^10,11^. Even incorporating p-NiO_x_ into Ga_2_O_3_ material system, the potential of Ga_2_O_3_ HJ PN diodes is only explored for less than 10% of the material limitation. Meanwhile, the conductivity modulation effect is observed in Ga_2_O_3_ HJ PN diodes, indicating the holes can be injected in the Ga_2_O_3_ layer^12^. However, under what bias condition and to what extent the conductivity modulation can impact the *R*_on,sp_ are still not explored. In addition, the in-depth understanding of bipolar transport in the Ga_2_O_3_ layer, especially hole transport and lifetime extraction are still forfeiting. Against the generally believed holes are self-trapped, the hole lifetime is a crucial and fundamental parameter to determine whether the bipolar transport is about to happen and to what extent it will impact the PN diode performances. Another critical issue regards the practical application of UWB PN diode is the requirement of low turn-on voltage (*V*_on_) for high-efficiency application, since the general forward bias (*V*_F_) is limited to be around 3 V. This is very challenging for homo-junction PN diode for wide bandgap semiconductor GaN and SiC with *V*_on_ ~ 3 V, regardless of the even wider bandgap Ga_2_O_3_.

In this article, a general design strategy of UWB semiconductor power diodes is provided to achieve high BV and low *R*_on,sp_ simultaneously through the introduction of hole injection and transport in Ga_2_O_3_ to minimize the *R*_on,sp_, suppressing the background carrier density to improve the BV, employing low conduction band offset p-NiO_*x*_ to reduce *V*_on_, and a composite E management technique with implanted ET and field plate architecture to further strengthen the BV. We setup a milestone of the UWB power diodes by acquiring a BV of 8.32 kV and P-FOM = BV^2^/*R*_on,sp_ of 13.21 GW/cm^2^, which is a record P-FOM value among all types of UWB power diodes to date, and it also exceeds the 1-D unipolar limit of GaN and SiC. Meanwhile, a conductivity modulation phenomenon induced bipolar transport of electron and hole pairs is identified with hole lifetime determined to be 5.4–23.1 ns. Considering some real application circumstances of diodes at a *V*_F_ of 3 V, benchmarking of the BV and *R*_on,sp_ extracted at *V*_F_ = 3 V also shows a record P-FOM value to date, validating the great promise of UWB power diodes for next-generation high-voltage and high-power electronics.

## Results and discussions

### High BV and low *R*_on,sp_ design strategy and implementation

The most intriguing aspect of β-Ga_2_O_3_ is that its native substrate can be substantially grown by the melt-grown methodology, which lays a basic foundation for low-cost and large-diameter with low defect density substrate^13^. The β-Ga_2_O_3_ epi-layer can be epitaxied by various routes, such as molecular beam epitaxy, metalorganic chemical vapor deposition (MOCVD), mist-CVD, halide vapor phase epitaxy (HVPE), and some other low-cost techniques^14,15^. HVPE is the most widely adopted methodology for balancing the epitaxial speed, substrate size, defect density, and complicity. The β-Ga_2_O_3_ background doping regulation is a challenge, resulting in a non-controllable electron density of 2–4 × 10^16^ cm^−3^. Unintentional doping from precursors like Si or H, and defects like O vacancies all contribute to the n-type conduction in the β-Ga_2_O_3_ layer.

Ideal power devices should embrace high BV and low *R*_on,sp_ to provide high blocking capability and low loss simultaneously^16^. In order to improve the BV of the UWB Ga_2_O_3_ power diodes, the minimal doping concentration is the first essential, since the slope of the E is governed by the doping concentration^16^. Summarized in Supplementary Fig. 1, it was found that the BV of the reported Ga_2_O_3_ power diodes is limited to be less than 3 kV, where the donor concentration is the major tyranny. Some other subsidiary factors like ETs or advanced E management techniques are all prerequisites for a minimized peak E at the anode edge to achieve a high desirable BV. PN junction is one of the most straightforward approaches to suppress the peak E at the interface. However, the forfeit of the p-Ga_2_O_3_ on the n-Ga_2_O_3_ makes the PN home-junction an impossible mission to further explore the maximum BV potentials of diodes. It should be noted that only extending the spacing of two electrodes to increase the BV is of marginal value by sacrificing the *R*_on,sp_ and averaged E. In terms of manipulating the *R*_on,sp_, to increase the doping concentration seems to be the simplest, however, the BV will be essentially compromised. A unique physical phenomenon of power diode, which is called conductivity modulation of the PN or PIN junction at forward bias will substantially guarantee a low *R*_on,sp_ even at a low doping concentration. Regardless of the challenge on the formation of PN homo-junction, the high *V*_on_ > 4 V is another suffering for the Ga_2_O_3_ homo-junction PN diodes.

Recently, the implementation of the p-NiO_x_ into the Ga_2_O_3_ system opens up another route for expanding the Ga_2_O_3_ application from the SBDs to the HJ PN diode^17–20^. Although the performance of the Ga_2_O_3_ HJ diode is still inferior to the SBDs at current stage, however, we argue that some fundamental limitations which have haunted the Ga_2_O_3_ power diodes research for a decade could be essentially clarified. First, p-NiO_x_ flavors a low conduction band offset of ~2.1 eV such that the high V_on_ issue of the homo-junction could be partially resolved. Second, with a PN HJ structure, the conductivity modulation is theoretically expected so that the R_on,sp_ can be minimized at a low doping concentration and high *V*_F_. In addition, by combining the ETs and advanced E management, the BV can be further enhanced. Comparison of the E management strategies is summarized in Supplementary Fig. 2.

**Fig. 1:**
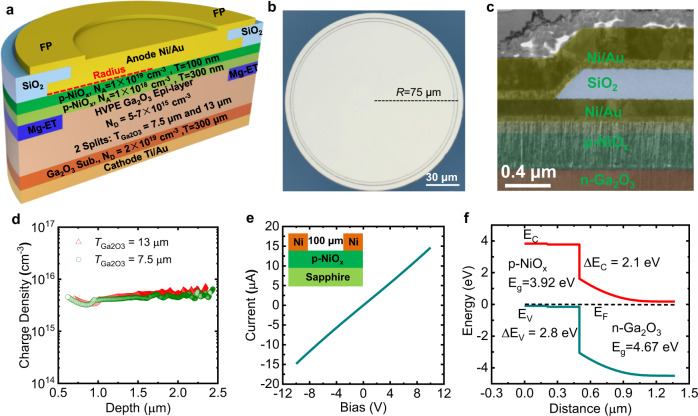
UWB power diodes design and implementation. **a** 3-D cross-sectional schematic of the Ga_2_O_3_ power diodes with HJ architecture and composite electric field management. **b** Top view of a fabricated Ga_2_O_3_ power diode. **c** False-colored SEM image of the cross-sectional anode field plate region with p-NiO_x_ thickness of 400 nm. **d** Extracted carrier concentration of two representative samples with concentration of 5 × 10^15^−7 × 10^15^ cm^−3^. **e** Current-voltage behavior of the Ni pads on p-NiO_x_ with *N*_A_ = 10^19^ cm^−3^, showing an Ohmic contact. **f** Simulated band diagram of the p-NiO_x_/n-Ga_2_O_3_ HJ structure. The band bending occurs in n-Ga_2_O_3_ and the conduction band offset is only 2.1 eV, showing the great promise of low *V*_on_ even for a UWB material.

Figure 1a shows the 3-D cross-sectional image of two representative Ga_2_O_3_ HJ PN diodes, the top view image is exhibited as Fig. 1b, and the false-colored scanning electron microscopy (SEM) image at the crucial area of the anode edge is listed in Fig. 1c. In the Ga_2_O_3_ power diodes, the doping concentration of the Ga_2_O_3_ epi-layer is suppressed from the 2 × 10^16^ cm^−3^ to around 5–7 × 10^15^ cm^−3^ for two wafers with different thicknesses by adopting a long duration of the oxygen thermal anneal process, as shown in Fig. 1d^21^. *C*–*V* curves are shown in Supplementary Fig. 3. Heavily doped p-NiO_x_ layer on top is utilized to form an Ohmic contact, as described in Fig. 1e. The simulated energy band diagram of the p-NiO_x_/*n*-Ga_2_O_3_ HJ is shown in Fig. 1f with the conduction band and valance band offset to be 2.15 eV and 2.8 eV, respectively. The ET process by Mg doping to form a high-resistivity region underneath the electrode is utilized to withstand a high E and the coupled field plate is implemented to further mitigate crowded peak E at the anode edge^15^.

### Diodes characterizations

**Fig. 2:**
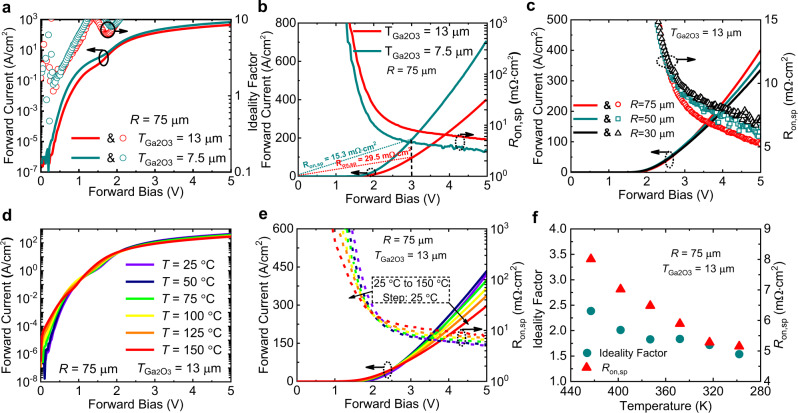
UWB Ga_2_O_3_ power diodes forward characteristics. **a** Forward current-voltage-ideality factor characteristics of two Ga_2_O_3_ power diodes with *T*_Ga2O3_ = 7.5 and 13 μm. **b** Forward current–voltage-specific on-resistance *R*_on,sp_ characteristics of the same diodes as **a**. A decent *V*_on_ = 1.8 V with minimal Diff. *R*_on,sp_ = 2.9 and 5.24 mΩ cm^2^ as well as extracted overall *R*_on,sp_ (@*V*_F_ = 3 V) of 15.3 and 29.5 mΩ cm^2^ for *T*_Ga2O3_ = 7.5 and 13 μm are achieved. **c** Radius-dependent forward current-voltage-resistance curves for diodes with *T*_Ga2O3_ = 13 μm. T-dependent **d** log-scale and **e** linear-scale forward characteristics of diode with *T*_Ga2O3_ = 13 μm. On/Off ratio of 10^10^ and 10^8^ are achieved for *T* = 25 °C and 150 °C, respectively. At all temperatures, *R*_on,sp_ drops when *V*_F_ increases, verifying the conductivity modulation effect. **f** Extracted T-dependent ideality factor and *R*_on,sp_ values as **e**.

Figure 2a compares the log-scale forward current-forward bias-ideality factor (*I*_F_-*V*_F_-*η*) characteristics of two Ga_2_O_3_ HJ PN diodes with Ga_2_O_3_ thickness (T_Ga2O3_) of 7.5 and 13 μm at a radius of 75 μm. The kink effect observed at *V*_F_ around 1.5 V is related to the variation of the barrier height and ideality factor, which is most likely to be induced by the two different barriers connected in parallel. *I*_F_ on/off ratio of 10^9^−10^10^ and *η* smaller than 2 can last for 4–5 decades of the *I*_F_. Figure 2b shows the linear-scale forward *I*_F_-*V*_F_-*R*_on,sp_ curves of the same diodes as Fig. 2a. Even with a PN HJ structure, a relatively decent *V*_on_ = 1.8 V is acquired, which is much smaller than the *V*_on_ of SiC and GaN PN diodes. The small *V*_on_ is benefited from two aspects, the small conduction band offset between p-NiO_x_ and n-Ga_2_O_3_ and the interface recombination current^17^. Minimal Diff. *R*_on,sp_ is extracted to be 2.9 and 5.24 mΩ cm^2^ for *T*_Ga2O3_ = 7.5 and 13 μm, respectively. Unlike SBDs with increased *R*_on,sp_ at an increased *V*_F_, the *R*_on,sp_ of the Ga_2_O_3_ HJ PN diodes drops at an increased *V*_F_, most likely due to bipolar transport-induced conductivity modulation effect. It should be noted that such conductivity modulation effect is the key to enable the simultaneous achievement of low *R*_on,sp_ and high BV. Figure 2c describes the radius-dependent *I*_F_-*V*_F_-*R*_on,sp_ curves for diodes with *T*_Ga2O3_ = 13 μm. Log-scale *I*_F_-*V*_F_ characteristic is summarized in Supplementary Fig. 4. By increasing the radius, the insulating Mg implanted region constitutes to a smaller portion of the area so that *R*_on,sp_ decreases when radius increases. The resistance (Res.) contribution from each layer based on the equation Res. = thickness/(*N*_D_ × *μ* × *q*) is summarized in Supplementary Fig. 5^22^. For diodes with *T*_Ga2O3_ = 7.5/13 μm, *N*_D_ = 6 × 10^15^ cm^−3^, *μ* = 200 cm^2^/Vs, and *q* = 1.6 × 10^−19^ C, the resistance of the drift layer is calculated to be 3.89/6.77 mΩ cm^2^. It should be noted that this calculation is based on the low-level injection prerequisite. At *V*_F_ = 5 V, conductivity modulation effect of the HJ PN diode begins to dominate so that the *R*_on,sp_ drops, which is favorable for resistance minimization. Figure 2d and  e summarizes the T-dependent linear-scale *I*-*V*-*R*_on,sp_ and log-scale *I*_F_-*V*_F_ of the diode with radius = 75 μm and *T*_Ga2O3_ = 13 μm. On/off ratios of 10^10^ and 10^8^ are achieved at *T* = 25 °C and 150 °C, respectively. At all temperature ranges, the differential (Diff.) *R*_on,sp_ drops at an increased *V*_F_, verifying the conductivity modulation effect of the Ga_2_O_3_ HJ PN diodes. Figure 2f shows the extracted T-dependent ideality factor *η* and *R*_on,sp_ from temperatures of 25–150 °C. The η is extracted from the forward current equation *J* = *J*_s_(exp(*qV*_F_/*ηkT*) − 1), whereas *J*_s_ is the reverse saturation current, V_F_ is the applied forward bias, *q* is the electron charge, k is the Boltzmann’s constant, and *T* is the absolute temperature. The *η* is extracted to be around 1.5 at *T* = 25 °C.

Based on the simulation, it is very interesting to find that hole concentration in the Ga_2_O_3_ layer at the HJ-interface is comparable with the Ga_2_O_3_ doping concentration of 6 × 10^15^ cm^−3^ at *V*_F_ = 3.5 V, as shown in Supplementary Figs. 6 and 7. That is to say, the hole injection-related conductivity modulation can help to reduce the *R*_on,sp_ only with *V*_F_ ≥ 3.5 V, since the hole is with more than 1 order of magnitude lower mobility. The simulation result is in good agreement with the forward *I*_F_–*V*_F_ characteristic, since the *R*_on,sp_ = 7 mΩ cm^2^ (at *V*_F_ = 3.5 V) roughly equals to the resistance summary of p-side Ohmic contact, p-NiO_x_ layer, n-Ga_2_O_3_ drift layer, n^+^-Ga_2_O_3_ substrate and n-side Ohmic contact. In other words, the hole injection and conductivity modulation are negligible at the *V*_F_ range of *V*_on_ = 1.8 V to 3.5 V, due to significant valance band offset between p-NiO_*x*_ and n-Ga_2_O_3_, so that few holes can be injected across this barrier. At *V*_F_ ~ 3.5 V, holes are injected from p-NiO_x_ to n-Ga_2_O_3_ most likely via trap assisted tunneling and hopping mechanisms. By increasing the V_F_ beyond 3.5 V to lower the PN HJ barrier, more holes are injected into n-Ga_2_O_3_ layer and hence high level injection phenomenon will raise the electron concentration in the Ga_2_O_3_ layer to maintain the charge neutrality condition. Therefore, the R_on,sp_ is further reduced when the *V*_F_ is increased. At *V*_F_ = 5 V, the hole concentration is simulated to be 3.8 × 10^16^ cm^−3^ and 6 × 10^15^ cm^−3^ at HJ-interface and 6 μm away from the HJ-interface, respectively. The averaged hole (also electron) concentration is extracted to be 1.9 × 10^16^ cm^−3^ within this 6-μm range, by integrating concentration and then divided by the total length of 6 μm. Therefore, the resistance of the significant hole injection region is roughly calculated to be 1.32 mΩ cm^2^, by considering the electron mobility of 150 cm^2^/Vs at this electron concentration. By adding up another 7-μm low level injected Ga_2_O_3_ layer resistance of 6.77/13 × 7 = 3.65 mΩ cm^2^, the 13-μm Ga_2_O_3_ drift layer owns a *R*_on,sp_ of 4.97 mΩ cm^2^. This estimation of the *R*_on,sp_ coincides with our extracted *R*_on,sp_ from the *I*_F_–*V*_F_, verifying the correctness of the explanation, hole concentration simulation, and calculation of the hole injection into the Ga_2_O_3_ layer.

**Fig. 3:**
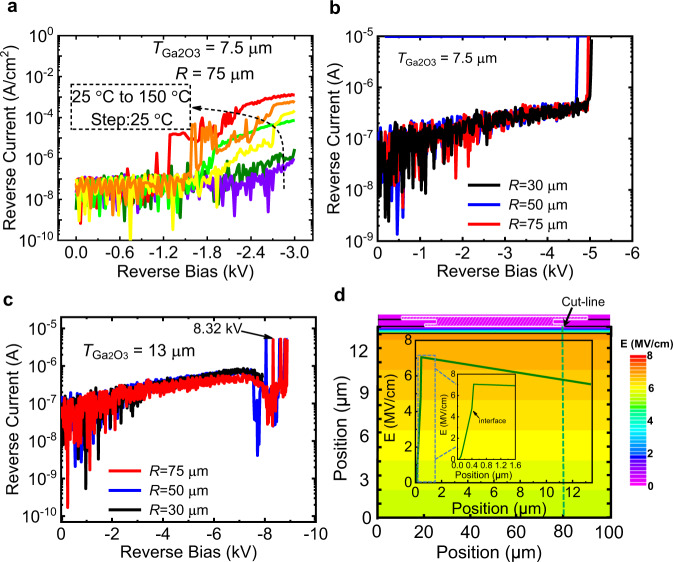
UWB Ga_2_O_3_ power diodes with high breakdown voltages. **a** T-dependent reverse current–voltage characteristics of diode with *T*_Ga2O3_ = 7.5 μm. With increased *T*, *I*_R_ increases, indicating a non-avalanche breakdown. Room temperature reverse current–voltage characteristics of diodes with *T*_Ga2O3_ = 7.5 μm (**b**) and 13 μm (**c**) at various radiuses. A BV of 5.1 kV and 8.32 kV are achieved for diodes with *T*_Ga2O3_ = 7.5 and 13 μm, yielding an averaged *E* of 6.45 MV/cm and 6.2 MV/cm, respectively. **d** Simulated E distribution of the diode with BV = 8.32 kV and T_Ga2O3_ = 13 μm. Due to the small *N*_D_ = 6 × 10^15^ cm^−3^, a fully depletion and a small E slope of the drift layer is observed.

The T-dependent reverse *I*–*V* characteristics of diode with *T*_Ga2O3_ = 7.5 μm are plotted in Fig. 3a from *T* = 25–150 °C. By increasing the *T*, *I*_R_ increases, indicating a non-avalanche breakdown behavior. Even at *T* = 150 °C, the *I*_R_ is just 1 mA/cm^2^ at a reverse bias of 3 kV. By further pushing the reverse bias to 5.1 kV we observe a hard breakdown with *T*_Ga2O3_ = 7.5 μm, as indicated in Fig. 3b. The averaged *E* field is calculated to be around 6.45 MV/cm by considering *E* = 5.1 kV/(0.4 μm + 7.5 μm). Combined with the *R*_on,sp_ = 2.9 mΩ cm^2^, the P-FOM = BV^2^/*R*_on,sp_ is yielded to be 8.97 GW/cm^2^. As for the diode with *T*_Ga2O3_ = 13 μm, a maximum BV of 8.32 kV is acquired at an *I*_R_ = 0.2 mA/cm^2^, as exhibited in Fig. 3c. The as-measured figure is shown in Supplementary Fig. 8. This BV = 8.32 kV is the highest BV value among all Ga_2_O_3_ power FETs and diodes to date. As a result, the P-FOM is calculated to be (8.32 kV)^2^/5.24 mΩ cm^2^ = 13.21 GW/cm^2^. Besides the record P-FOM, this HJ PN diode also has a high averaged *E* = 8.32 kV/(0.4 μm + 13 μm) = 6.2 MV/cm. Figure 3d describes the E simulation result of the HJ PND with *T*_Ga2O3_ = 13 μm and BV = 8.32 kV. The simulated peak E in the p-NiO_x_ layer is around 4.9 MV/cm, which is slightly lower than its theoretical limit, considering the 3.9 eV bandgap. The peak E at the p-NiO_x_ side is lower when compared with the peak *E* at the Ga_2_O_3_ side, due to much higher dielectric constant of p-NiO_x_. Due to the small *N*_D_ and the depletion effect from the p-NiO_x_ as well as the functionalities of the ET and coupled field plate, a fully depletion and small E slope are observed in the drift layer, resulting a peak *E* = 7 MV/cm in the Ga_2_O_3_ at the HJ-interface.

### Holes in Ga_2_O_3_ layer

Similar to other UWB semiconductors like diamond, BN, and AlN, high ionization efficiency of n- and p-type doing simultaneously turns out to be a big challenge, considering the UWB nature of those UWB semiconductor materials. The direct observation of conductivity modulation is a straightforward evidence of bipolar transport and hole existence in the Ga_2_O_3_ layer, which deviates from the general prediction that holes are less likely to occur in Ga_2_O_3_. Three reasons are attributed to the challenge of acquiring holes in Ga_2_O_3_, no calculated shallow acceptors, large effective mass from the flat valance band, and free holes tend to be self-trapped by polarons. However, we argue that with the unique PN HJ structure under high *V*_F_ condition, holes from the heavily-doped p-NiO_x_ are capable of being injected to the Ga_2_O_3_ layer, although the hole mobility is relatively low. Under a very positive *V*_F_ condition (e.g., 5 V), energy band of the p-NiO_x_ is pulled down so that holes at the Fermi tail witness no significant barrier height to travel across the PN HJ-interface and then diffuse in the Ga_2_O_3_ layer, leading to the conductivity modulation effect. In other words, the holes can be manufactured in the UWB Ga_2_O_3_ layer by hole injection at a very positive *V*_F_. In order to verify the hole transportation and survival in the Ga_2_O_3_ not so short by self-trapping effect of the polarons, hole lifetime extraction or measurement is urgently needed.

**Fig. 4:**
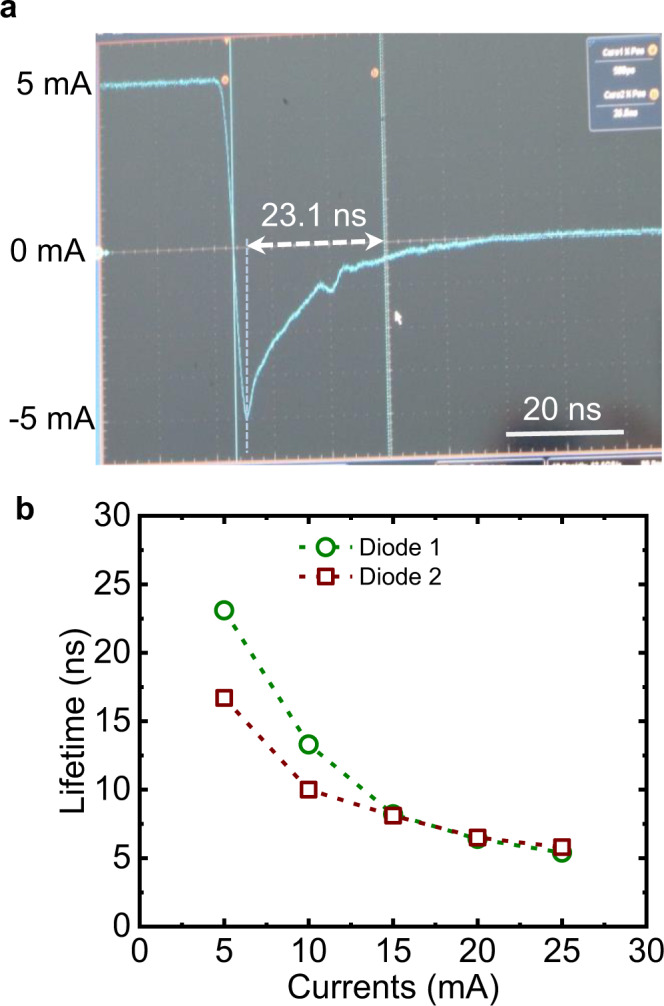
Hole lifetime determination in Ga_2_O_3_ layer. **a** Time-dependent of the reverse recovery characteristics of Ga_2_O_3_ HJ PN diode at a forward injection current of 5 mA. **b** Lifetime dependence on the forward injection current with current ranges from 5 mA to 25 mA. At current of 5 mA, the lifetime is determined to be 23.1 ns. At high *V*_F_ condition, holes diffuse from p-NiO_x_ to n-Ga_2_O_3_ without seeing obvious barrier, so that the hole lifetime in the Ga_2_O_3_ layer can be determined.

The reverse recovery measurement technique is implemented to determine the hole lifetime in the Ga_2_O_3_ layer, and the schematic of the measurements are summarized in Supplementary Fig. 9a^23^. Once the Ga_2_O_3_ diode is switched from positive *V*_F_ to a reverse bias, a period of time is needed to remove holes from the Ga_2_O_3_ either via electron-hole pair recombination or to be trapped by polarons. The hole lifetime (*τ*_p_) can be determined by the equation *τ*_p_ = *t*_sd_/(erf^−1^(*I*_F_/(*I*_F_ + *I*_R_)))^2^, whereas *t*_sd_, *I*_F_, and *I*_R_ represent charge storage time, forward current, and reverse current, respectively^24^. During the reverse recovery measurement, the *V*_F_ is extracted to be 2.97 V and 4.73 V for injection current of 5 mA and 25 mA, respectively, at a diode radius of 40 μm. Meanwhile, the subsequently applied reverse bias is −8 V. The reverse recovery and input–output measurement of the pulsed current–voltage characteristics of the UWB Ga_2_O_3_ HJ PN diode at a diode current of 5 mA is shown in Fig. 4a. For the HJ PN diode, the *I*_F_ is 5 mA which is 3 orders of magnitudes more than *I*_R_, so that *I*_F_/(*I*_F_ + *I*_R_) can be simplified to be 1. Then the *τ*_p_ can be simplified to *π*/4 × *t*_sd_, which is ~80% of the *t*_sd_ when the diode is switched until the anode current is recovered to be around 0. Therefore, hole lifetime *τ*_p_ is determined to be 23.1 ns at a forward injection current *I*_F_ of 5 mA. The *τ*_p_ dependence on *I*_F_ is summarized in Fig. 4b, with a minimal *τ*_p_ of 5.4 ns. In order to exclude the subsidiary impact on the measurements, the reverse recovery measurement is performed on Ga_2_O_3_ SBD (Supplementary Fig. 9b) and the recovery time in the SBD is determined to be 1.8 ns, which is 1 order of magnitude lower when compared with the HJ PN diode. By injecting holes into the Ga_2_O_3_ layer at high *V*_F_, the hole lifetime is then determined to be 5.4–23.1 ns. By combining the calculated hole effective mass (*m*_p_*) of 4.46*m*_o_ (Supplementary Fig. 10), the hole mobility (*μ*_p_) can be roughly estimated by the equation *μ*_p_ = *q* × *τ*_p_/*m*_p_*, yielding the *μ*_p_ to be 1.93–8.3 cm^2^/Vs.

### Performance benchmarking

**Fig. 5:**
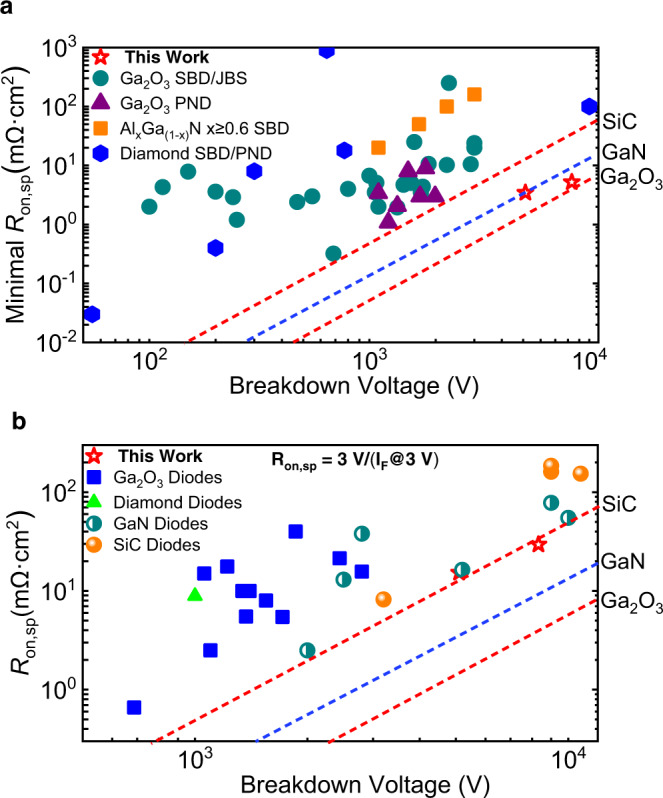
Benchmarking UWB Ga_2_O_3_ power diodes against state-of-the-art other diodes. **a** Minimal *R*_on,sp_ versus BV of some representative UWB power diodes, including Ga_2_O_3_, diamond, and high-Al AlGaN, which are reported in the literatures. Our Ga_2_O_3_ power diodes set a milestone for the UWB power diodes by breaking the 1-D unipolar figure-of-merit limit of GaN and SiC. **b** Extracted *R*_on,sp_(@3V) versus BV of some highest performance GaN, SiC, and diamond diodes. By considering some real application circumstances, the *R*_on,sp_ = 3V/(I_F_@3 V) is preferred over the minimal *R*_on,sp_ to eliminate the impact of the *V*_on_. GaN and SiC PN diodes are excluded due to the *V*_on_ ~ 3 V. Our UWB power diodes demonstrate a substantial enhancement of the performance over other diodes by surpassing the 1-D unipolar limit of the SiC. The *R*_on,sp_ extraction for lateral diodes is yielded by *R*_on,sp_ = on-resistance × (anode–cathode spacing +1.5 μm transfer length for both electrodes).

The combination of the conductivity modulation induced low *R*_on,sp_ and low doping concentration as well as the composite E regulation led record BV renders a substantial performance enhancement by setting a record P-FOM of all UWB power diodes (Fig. 5a), including Ga_2_O_3_, diamond, and high Al-Al_*x*_Ga_1−*x*_N (*x* > 60%) power diodes^25–44^. Compared with all other Ga_2_O_3_ power diodes, the BV of this work is around three times the previously reported best BV of 2.9 kV with a lower *R*_on,sp_. The most enticing aspect of this work is that the performance of the Ga_2_O_3_ device exceeds the 1-D unipolar limit of the SiC and GaN. In terms of real application of the HJ PN diode in the circuit, the overall *R*_on,sp_ at a general *V*_F_ = 3 V instead of the minimal differential *R*_on,sp_ is more realistic. In order to eliminate the impact of *V*_on_, an overall *R*_on,sp_ of 15.3 mΩ cm^2^ and 29.5 mΩ cm^2^ are extracted for *T*_Ga2O3_ of 7.5 μm and 13 μm at a *V*_F_ = 3 V, respectively, as shown in Fig. 2b. Benchmarking against all other state-of-the-art representative diodes, including SiC SBDs/JBS diodes/PN diodes and GaN SBDs/PN diodes with extracted *R*_on,sp_ at a fixed *V*_F_ = 3 V, our Ga_2_O_3_ HJ PN diodes achieve a record of nowadays power diodes, as compared in Fig. 5b^33,45–53^. Even under the real application circumstance, the P-FOM = BV^2^/*R*_on,sp_ of the Ga_2_O_3_ power diodes still surpasses the 1-D unipolar limit of the SiC. These intriguing results verify the great promise of UWB semiconductor Ga_2_O_3_ power diodes for next-generation high-voltage and high-power electronics.

In summary, we show that UWB semiconductor Ga_2_O_3_ power diodes are capable of delivering a record high BV^2^/*R*_on,sp_, which breaks the 1-D unipolar limit of the SiC and GaN figure-of-merit. The incorporation of suppressed background doping, HJ PN structure, and the composite electric field management technique yields a high BV which makes the averaged electric field approach the material limit. Taking advantage of the hole injection as well as the conductivity modulation, the *R*_on,sp_ can be essentially minimized even the Ga_2_O_3_ is with a low doping concentration. The hole lifetime is determined to be 5.4–23.1 ns, which verifies the existence of the hole in the Ga_2_O_3_ layer. By carefully engineering the energy band offset, a decent *V*_on_ can also be derived for a high efficiency rectifying. This unique technology by implementing the low doping material, electric field suppression, hole injection as well as the conductivity modulation, and energy band engineering offers an effective route for the innovation of other UWB power diodes, such as diamond, BN, high Al mole fraction Al_x_Ga_1-x_N.

## Methods

### Fabrication of UWB Ga_2_O_3_ power diodes

Ga_2_O_3_ epi-wafers with epi-layer thicknesses of 7.5 μm and 13 μm were epitaxial by HVPE on a (001) substrate with substrate doping concentration of 2 × 10^19^ cm^−3^. Substrates were first thinned down from 650 μm to 300 μm by polishing to minimize on-resistance. Then, Ga_2_O_3_ epi-wafers were annealed in the low-pressure-CVD furnace at 500 °C under the O_2_ ambient to partially compensate the donors in the epi-layer. N-side Ohmic contacts were formed by evaporating Ti/Au metals followed with rapid thermal anneal at 450 °C. Angle-dependent Mg ion implantation was utilized to form a high-resistivity layer to serve as the ET. Bi-layers of p-NiO_x_ were sputtered at room temperature with first and second layer doping concentration of 1 × 10^18^ cm^−3^ and 1 × 10^19^ cm^−3^, respectively. The doping concentration of the p-NiO_x_ layer was confirmed by the Hall measurements and the Hall mobility of the second p-NiO_x_ layer is 1.1 cm^2^/Vs. P-side Ohmic contacts were formed by depositing Ni/Au layers. The field plate was constructed by depositing 300 nm of SiO_2_, SiO_2_ etching, and field plate metal evaporation. A summary of the device process schematic flow is shown in Supplementary Fig. 11.

### Device characterizations

The forward *I*–*V* and *C*–*V* characteristics were carried out by the Keithley 4200 semiconductor analyzer systems. Reverse *I*–*V* measurements were performed by Agilent B-1505A high voltage semiconductor analyzer systems with extended high-voltage module up to 10 kV. The hole lifetime measurements were carried out by reverse recovery measurement methods as Supplementary Fig. 9.

## Supplementary information

Supplementary Information

Peer Review File

## Data Availability

The data that support the plots within this paper and other findings of this study are available from the corresponding author upon reasonable request. The reason for controlled access is due to privacy issue. The data is available from the corresponding author H.Z. for research purposes and the corresponding author will send the data within one week once received the request.

## Code availability

The simulation code that supports the plots within this paper and other findings of this study are available from the corresponding author upon reasonable request. The reason for controlled access is due to privacy issue. The data are available from the corresponding author H.Z. for research purposes and the corresponding author will send the data within one week once received the request.
