# Supplementary material for: Ultra-wide bandgap semiconductor Ga2O3 power diodes
Source: Nat Commun. 2022 Jul 6;13:3900. doi: 10.1038/s41467-022-31664-y (PMC9259626; doi:10.1038/s41467-022-31664-y)
Supplement: Supplementary file 1 — Supplementary Information [file 41467_2022_31664_MOESM1_ESM.pdf]

# Supplementary Information

## Ultra-Wide Bandgap Semiconductor $Ga_2O_3$ Power Diodes

Jincheng Zhang[1], Pengfei Dong[1], Kui Dang[1], Yanni Zhang[1], Qinglong Yan[1], Hu Xiang[1], Jie Su[1], Zhihong Liu[1], Mengwei Si[2], Jiacheng Gao[3], Moufu Kong[3], Hong Zhou[1*], Yue Hao [1]

1 State Key Discipline Laboratory of Wide Bandgap Semiconductor Technology, School of Microelectronics, Xidian University, Xi'an 710071, China

2 Department of Electronic Engineering, Shanghai Jiao Tong University, Shanghai 200240, China.

3 State Key Laboratory of Electronic Thin Films and Integrated Devices of China, University of Electronic Science and Technology of China, Chengdu 61005.

[*]Correspondence to: hongzhou@xidian.edu.cn

1. Doping concentration versus breakdown voltage

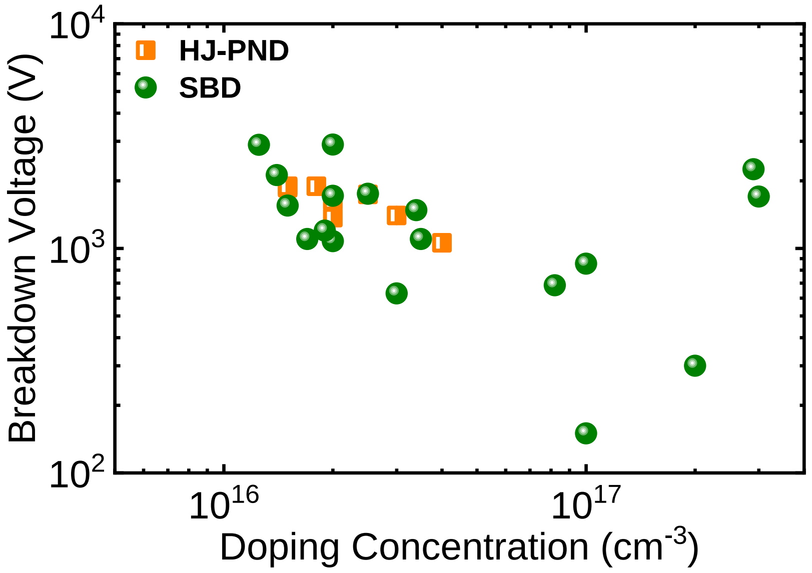


**Supplementary Figure 1.** Breakdown voltage versus doping concentration of UWB $Ga_2O_3$ power diodes, which are reported in the literatures. The maximum BV is generally constrained to be around 3 kV due to the high $N_d$ and non-optimized ET techniques.

## 2. Comparison of various electric field management strategies for BV improvement

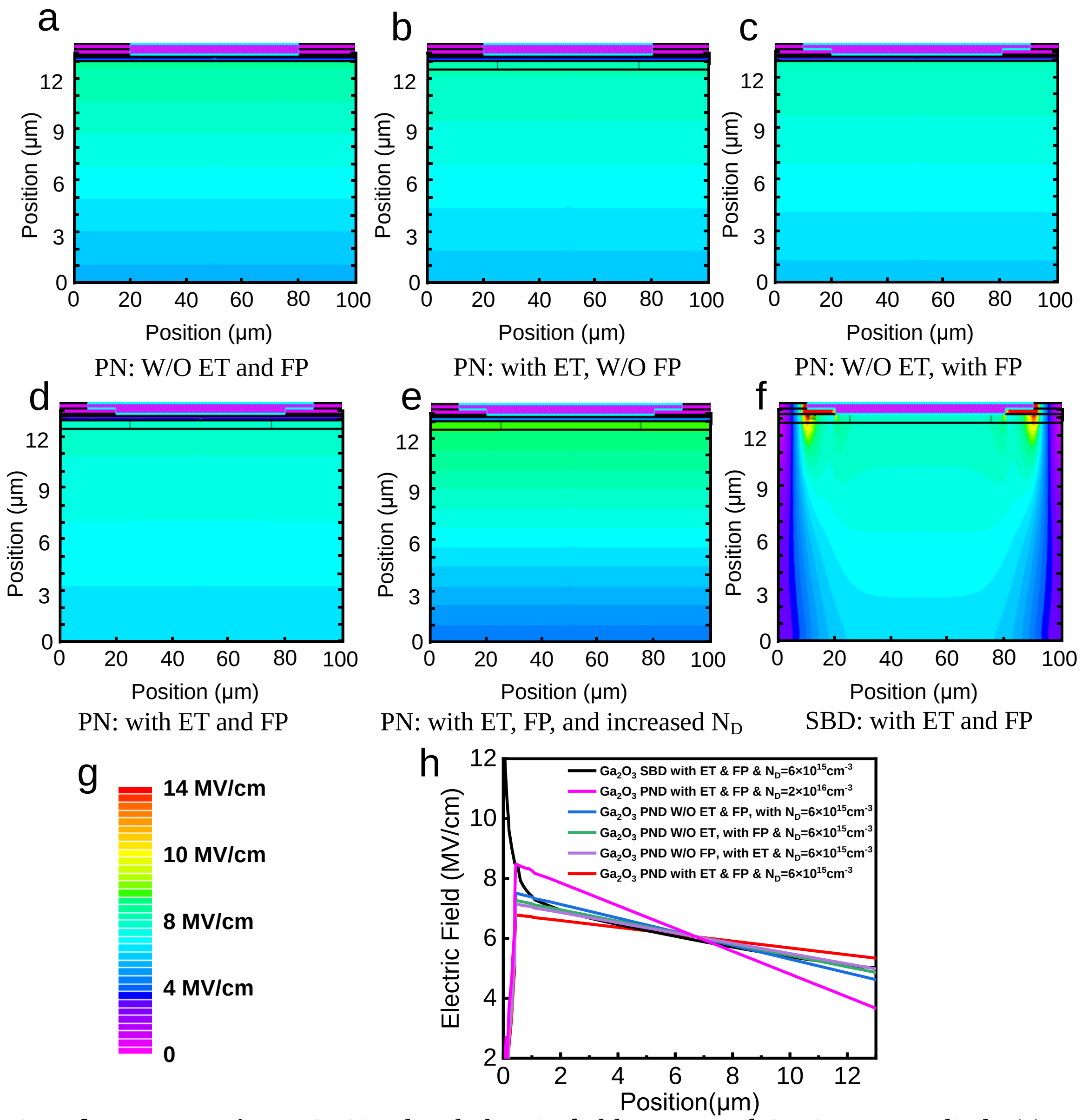


**Supplementary Figure 2.** Simulated electric field contour of $Ga_2O_3$ HJ-PN diode (a) without Mg-implanted ET and FP, (b) with Mg-implanted ET but without FP, (c) with FP but without Mg-implanted ET, (d) with Mg-implanted ET and FP at a doping concentration of $6\times10^{15}$ $cm^{-3}$. (e) $Ga_2O_3$ HJ-PN diode with Mg-implanted ET, FP and doping concentration of $2\times10^{16}$ $cm^{-3}$.(f) $Ga_2O_3$ SBD with Mg-implanted ET, FP and doping concentration of $6\times10^{15}$ $cm^{-3}$.(g) Electric field scale-bar. (h)Extracted E-field at anode edge for (a)-(f) structures. With the same doping concentration, diode structures (ET and FP), and BV = 8.3 kV, PN diode has a lower electric field. Minimizing the doping concentration can essentially reduce electric field. Implanted ET and FP can both reduce the electric field and their functionalities can be superimposed to each other to further suppress electric field.

3. C-V measurements of the annealed diodes

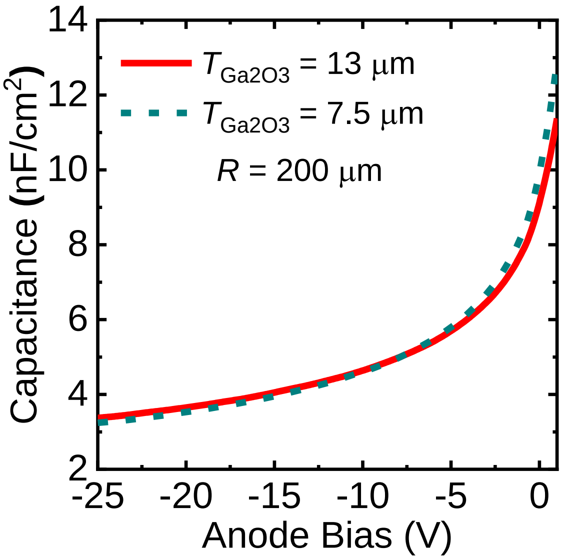


**Supplementary Figure 3.** Measured C-V curves of annealed two low doping samples with $T_{Ga2O3}$ = 7.5 and 13 μm.

4. Radius dependent log-scale forward I-V characteristics.

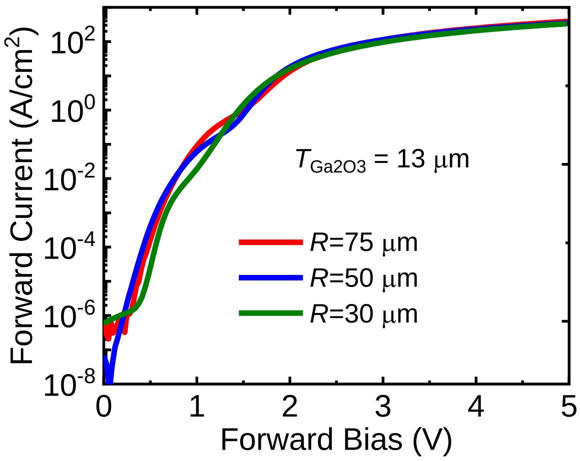


**Supplementary Figure 4.** Radius dependent log-scale forward I-V characteristics.

## 5. Resistance distribution

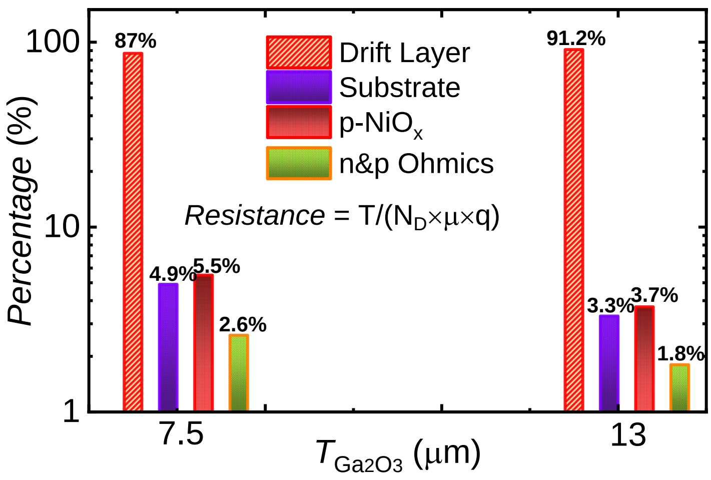


**Supplementary Figure 5.** Calculated components of the $R_{on,sp}$ with $T_{Ga2O3}$ = 7.5 and 13 μm by Res.=T/($N_D$×μ×q) by considering the low-level injection prerequisite. Drift layer occupies 91.2% of $R_{on,sp}$ for $T_{Ga2O3}$ = 13 μm. By combining the conductivity modulation effect, the drift layer resistance can be essentially reduced.

6. Band diagram simulation at forward bias conditions.

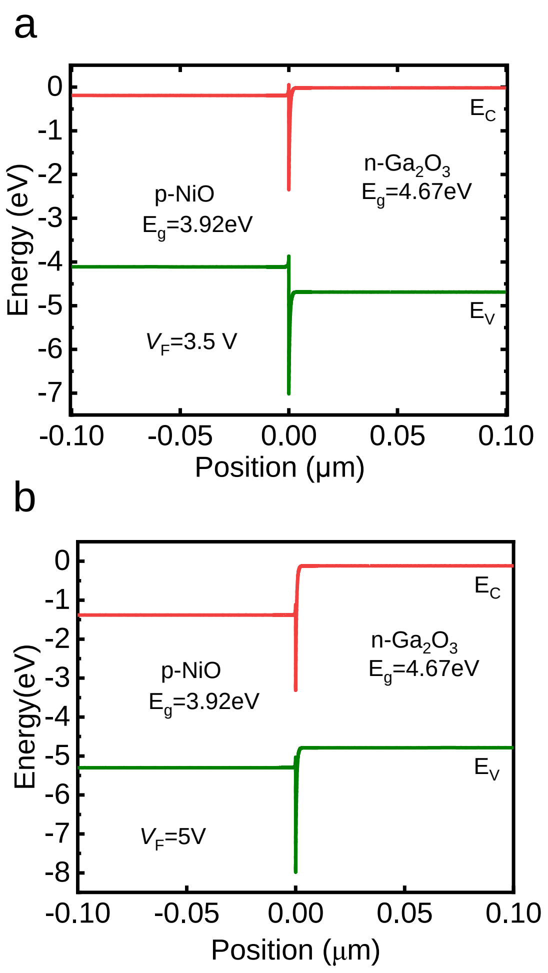


**Supplementary Figure 6.** Simulated conduction band and valance band under forward bias of (a) 3.5 V and (b) 5 V conditions.

## 7. Hole concentration simulation under various forward bias conditions

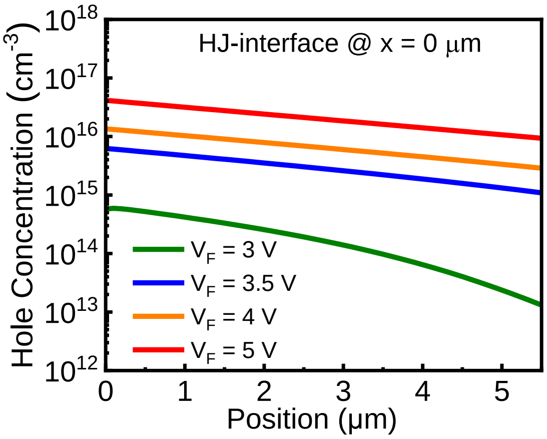


**Supplementary Figure 7.** Simulated hole concentration in the $Ga_2O_3$ layer under various forward bias conditions. During the simulation, heterojunction trap assisted tunneling and hopping, band to band tunneling, thermionic emission, Auger recombination, SRH recombination, and direct tunneling models are implemented. $Ga_2O_3$ minority carrier trap states with 0.1-0.7 eV above $E_V$ and density of $10^{16}$-$10^{17}$ $cm^{-3}$ are imposed and assumed in the model. At $V_F$ = 3 V, the hole concentration is extracted to be $5.6\times10^{14}$ $cm^{-3}$, which is negligible when compared with electron concentration in the $Ga_2O_3$ layer. The hole injection contribution to $R_{on,sp}$ suppression can be neglected since the hole mobility is more than one order of magnitude lower when compared with electron mobility. At $V_F$ = 3.5 V, the hole concentration at the HJ-interface is extracted to be around $6\times10^{15}$ $cm^{-3}$, such that high level injection occurs. By further increasing the $V_F$, more holes are injected in the $Ga_2O_3$ layer and essentially the electron concentration is increased, in order to maintain charge neutrality in the $Ga_2O_3$ layer. Therefore, the $R_{on,sp}$ can be substantially reduced by the conductivity modulation as well as the hole high level injection.

## 8. As measured breakdown figures

a

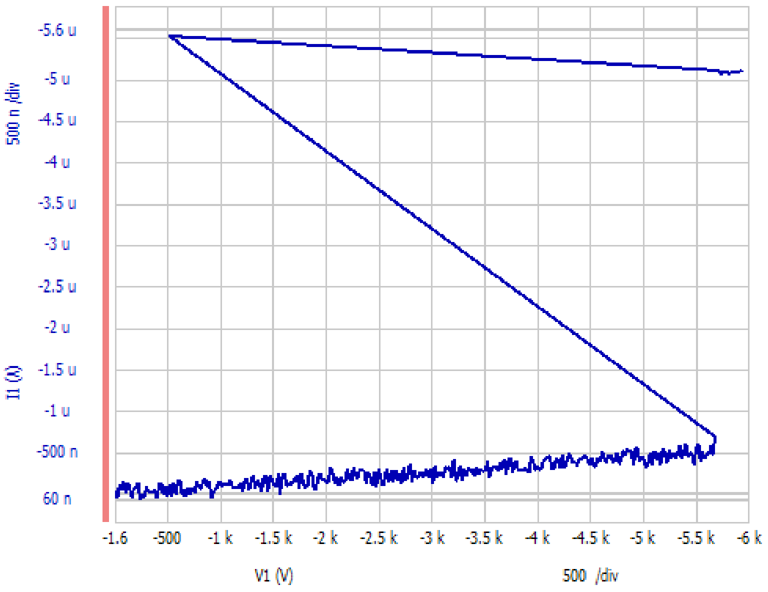


b

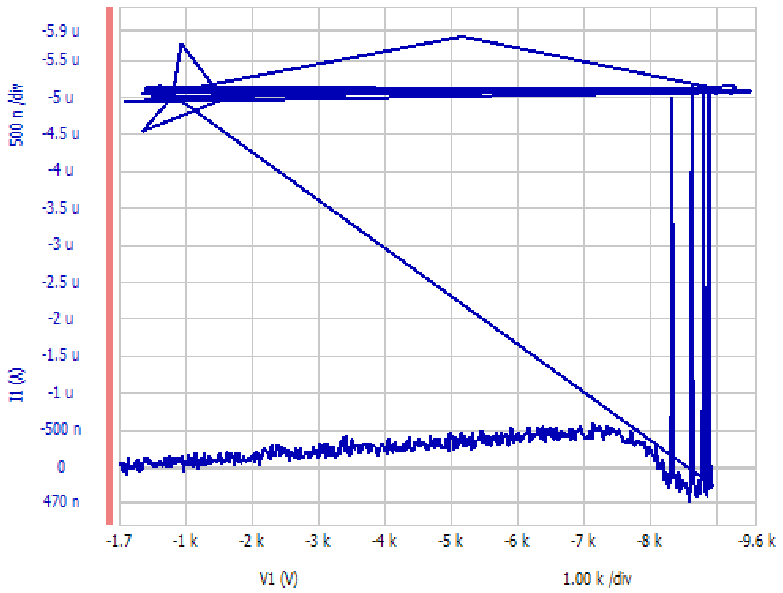


**Supplementary Figure 8.** As-measured figures for UWB $Ga_2O_3$ power diodes with BV = 5.65 kV and 8.32 kV at $T_{Ga2O3}$ = 7.5 µm (a) and 13 µm (b), respectively.

9. Reverse recovery measurements

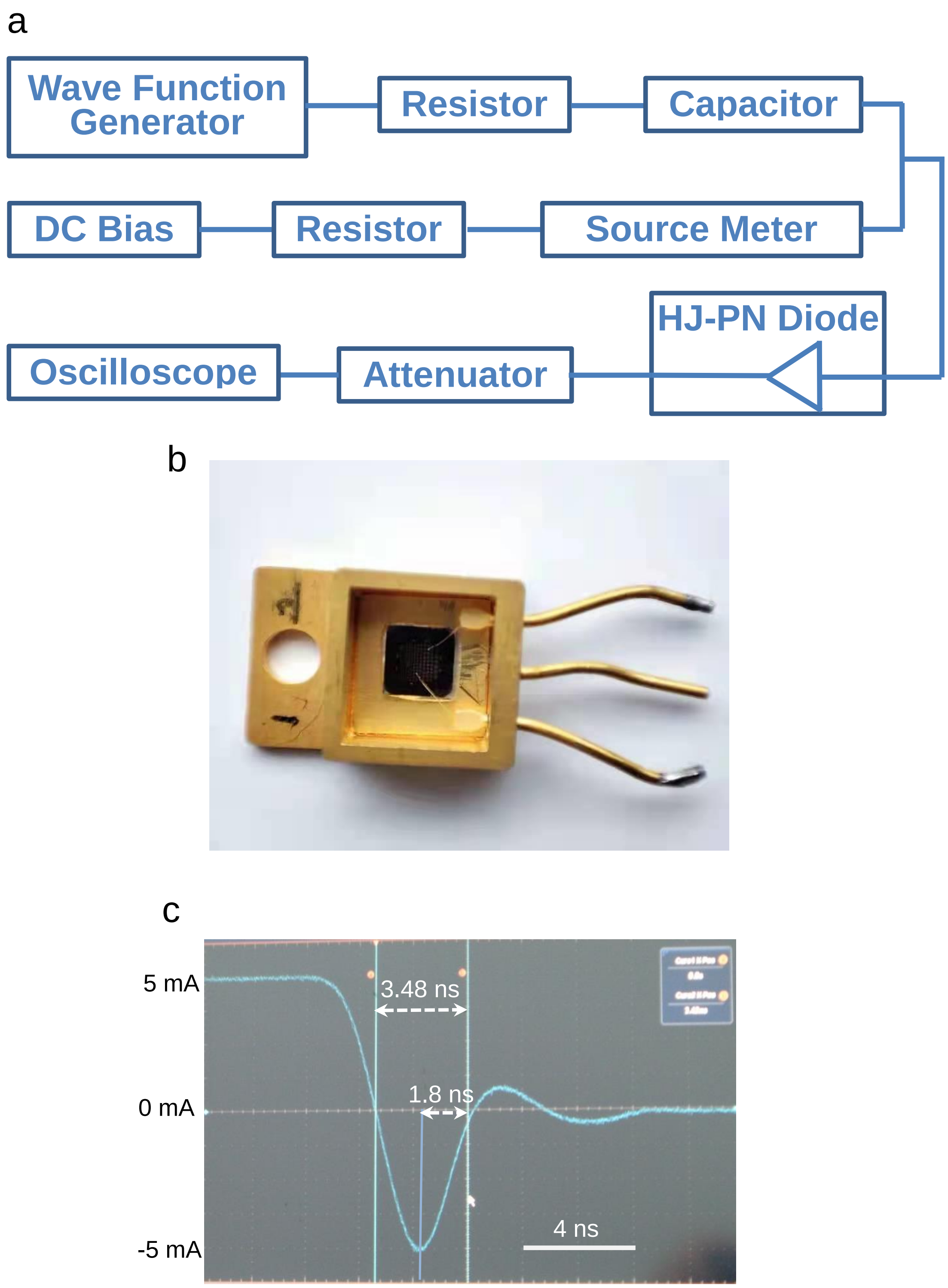


**Supplementary Figure 9.** (a) Reverse recovery measurements set-up schematic for hole lifetime measurement. (b) Packaged $Ga_2O_3$ HJ-PN diodes for reverse recovery measurements. (c) Reverse recovery measurements of $Ga_2O_3$ SBD with 1.8 ns lifetime.

10. $Ga_2O_3$ band structure and density of states

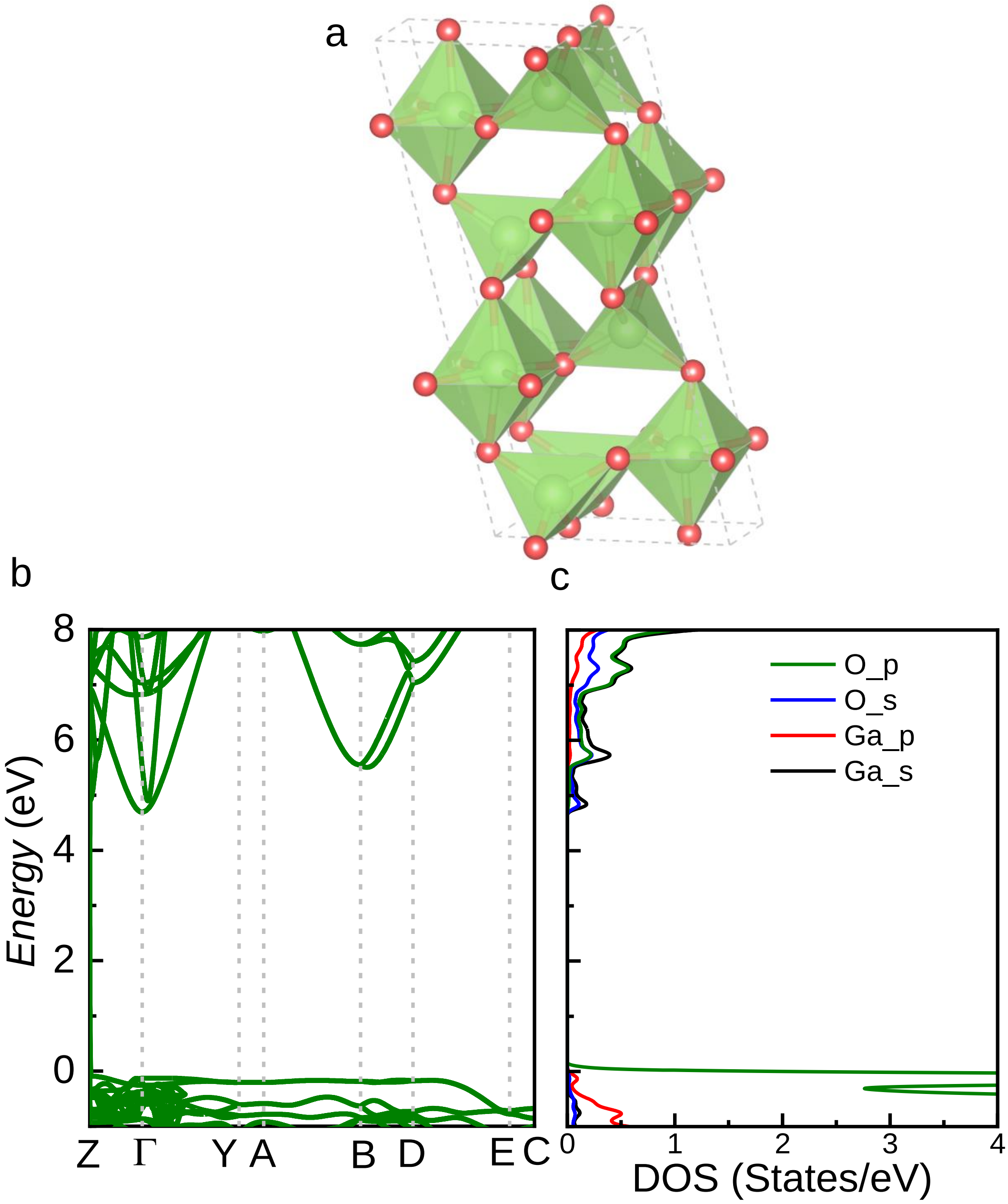


**Supplementary Figure 10.** (a) As-constructed atomic configuration of β-$Ga_2O_3$ crystal. (b) Calculated energy band structure and (c) calculated density of states (DOS) of bulk β-$Ga_2O_3$ crystal. The hole effective mass is extracted to be 4.46 $m_o$. Combing the hole lifetime of 5.4~23.1 ns, the hole mobility is roughly calculated to be 1.93~8.3 $cm^2$/Vs.

## 11. Device fabrication process flow

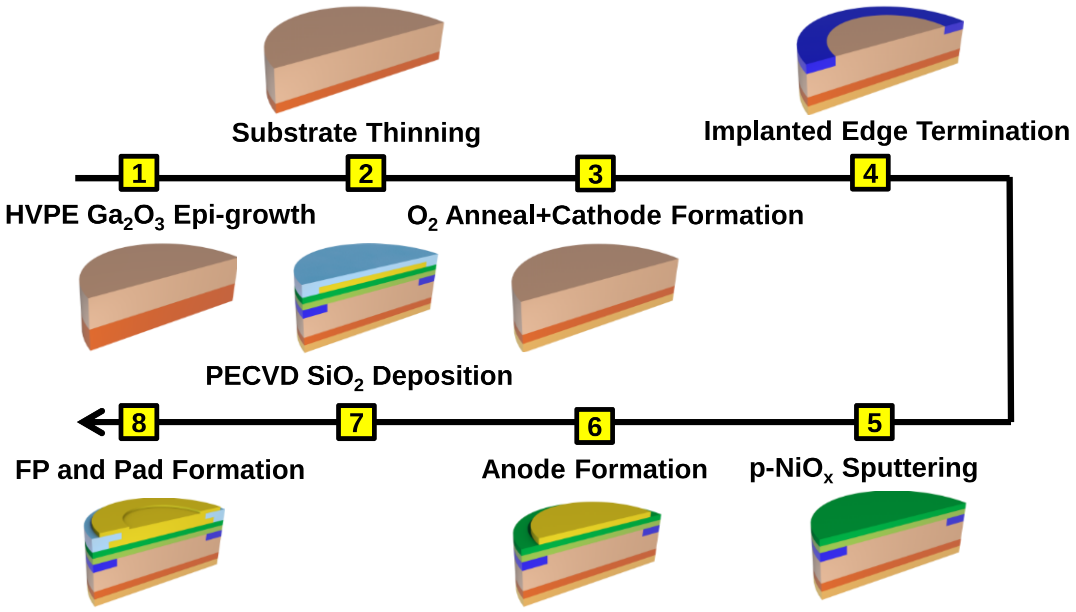


**Supplementary Figure 11.** Main fabrication steps of the $Ga_2O_3$ HJ PNDs. Some major processes include substrate thinning, $O_2$ thermal anneal process, Mg implanted edge termination to form isolation, double layer p-$NiO_x$ sputtering and FP formation. $O_2$ thermal anneal is used to minimize background dopant concentration, and the implantation as well as field plate are utilized as the composite electric field management to mitigate the crowded electric field at the anode edge.

## 12. Comparisons of SiC, GaN and $Ga_2O_3$ power semiconductor materials, and comparisons of p-type $NiO_x$ and $Cu_2O$

Table 1: Comparisons of SiC, GaN and $Ga_2O_3$ power semiconductor materials

| Materials | 4H-SiC | GaN | $\beta$-$Ga_2O_3$ |
|---|---|---|---|
| Bandgap $E_g$ (eV) | 3.25 | 3.4 | 4.8 |
| Dielectric Constant $\varepsilon$ | 10 | 9 | 9-10 |
| Breakdown Field $E_C$ (MV/cm) | 2.5 | 3.3 | 8 |
| Electron Mobility $\mu$ ($cm^2/Vs$) | 1000 | 1200 | 300 |
| Saturation Velocity $\nu_{sat}$ ($10^7$ cm/s) | 2 | 2.5 | 2 |
| Thermal Conductivity $\kappa$ (W/mK) | 370 | 250 | 10-30 |
| FOM relative to Si | | | |
| Baliga FOM $=\varepsilon\times\mu\times E_c^3$ | 317 | 846 | 3200 |
| Johnson FOM $= E_c^2\times\nu_{sat}^2/4\pi^2$ | 278 | 1089 | 2844 |
| Baliga High Frequency FOM $=\mu\times E_c^2$ | 46 | 100 | 142 |
| Keyes FOM $= \kappa\times[(c\times\nu_{sat})\times(4\pi\times\varepsilon)]^{1/2}$ | 3.6 | 1.8 | 0.2 |

Table 2: Comparisons of p-$NiO_x$ and p-$Cu_2O$

| Materials | $NiO_x$ | $Cu_2O$ |
|---|---|---|
| Bandgap $E_g$ (eV) | 3.7-4 | ~3 |
| Dielectric Constant $\varepsilon$ | 11.9 | 8.27 |
| Breakdown Field $E_C$ (MV/cm) | 4.3-5 | ~2.8 |
| Hole Mobility $\mu$ ($cm^2/Vs$) | 5 | 2.7 |
